# 3,5-Bis(4-fluoro­phen­yl)-4,5-dihydro-1*H*-pyrazole-1-carbaldehyde

**DOI:** 10.1107/S160053681101587X

**Published:** 2011-05-07

**Authors:** Zeliha Baktır, Mehmet Akkurt, S. Samshuddin, B. Narayana, H. S. Yathirajan

**Affiliations:** a Department of Physics, Faculty of Sciences, Erciyes University, 38039 Kayseri, Turkey; b Department of Studies in Chemistry, Mangalore University, Mangalagangotri 574 199, India; c Department of Studies in Chemistry, University of Mysore, Manasagangotri, Mysore 570 006, India

## Abstract

In the title mol­ecule, C_16_H_12_F_2_N_2_O, the pyrazole ring adopts a slight envelope conformation with the methyl­ene C atom deviating by 0.114 (3) Å from the mean plane of the other four atoms [maximum deviation = 0.021 (3) Å]. The dihedral angles between the four essentially planar atoms of the pyrazole ring and the fluoro-substituted benzene rings are 2.6 (2) and 82.2 (2)°. The dihedral angle between the two benzene rings is 83.7 (2)°. The crystal packing is stabilized by weak inter­molecular C—H⋯O hydrogen bonds.

## Related literature

For the biological activity of pyrazolines, see: Hes *et al.* (1978 ▶); Manna *et al.* (2005 ▶); Amir *et al.* (2008 ▶); Regaila *et al.* (1979 ▶); Sarojini *et al.* (2010 ▶). For their importance in organic synthesis, see: Bhaskarreddy *et al.* (1997 ▶); Klimova *et al.* (1999 ▶). For related structures, see: Butcher *et al.* (2007 ▶); Cui & Li (2010 ▶); Fun *et al.* (2010*a* ▶,*b* ▶); Jasinski *et al.* (2010*a* ▶,*b* ▶); Baktır *et al.* (2011 ▶).
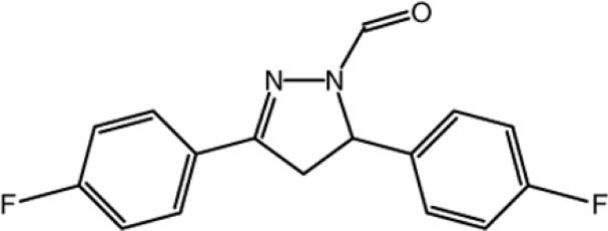


## Experimental

### 

#### Crystal data

C_16_H_12_F_2_N_2_O
*M* *_r_* = 286.28
Triclinic, 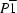

*a* = 6.2141 (9) Å
*b* = 6.7802 (8) Å
*c* = 17.9857 (9) Å
α = 96.727 (4)°
β = 90.254 (4)°
γ = 116.791 (5)°
*V* = 670.39 (13) Å^3^
*Z* = 2
Mo *K*α radiation
μ = 0.11 mm^−1^
*T* = 294 K
0.30 × 0.20 × 0.10 mm

#### Data collection

Rigaku R-AXIS RAPID-S diffractometer
Absorption correction: multi-scan (Blessing, 1995 ▶) *T*_min_ = 0.968, *T*_max_ = 0.989
14070 measured reflections
2736 independent reflections
1011 reflections with *I* > 2σ(*I*)
*R*_int_ = 0.095

#### Refinement

*R*[*F* ^2^ > 2σ(*F* ^2^)] = 0.062
*wR*(*F* ^2^) = 0.206
*S* = 0.94
2736 reflections
191 parameters
H-atom parameters constrained
Δρ_max_ = 0.19 e Å^−3^
Δρ_min_ = −0.33 e Å^−3^

### 

Data collection: *CrystalClear* (Rigaku/MSC, 2005 ▶); cell refinement: *CrystalClear*; data reduction: *CrystalClear*; program(s) used to solve structure: *SIR97* (Altomare *et al.*, 1999 ▶); program(s) used to refine structure: *SHELXL97* (Sheldrick, 2008 ▶); molecular graphics: *PLATON* (Spek, 2009 ▶); software used to prepare material for publication: *WinGX* (Farrugia, 1999 ▶).

## Supplementary Material

Crystal structure: contains datablocks global, I. DOI: 10.1107/S160053681101587X/lh5239sup1.cif

Structure factors: contains datablocks I. DOI: 10.1107/S160053681101587X/lh5239Isup2.hkl

Supplementary material file. DOI: 10.1107/S160053681101587X/lh5239Isup3.cml

Additional supplementary materials:  crystallographic information; 3D view; checkCIF report

## Figures and Tables

**Table 1:** Hydrogen-bond geometry (Å, °)

*D*—H⋯*A*	*D*—H	H⋯*A*	*D*⋯*A*	*D*—H⋯*A*
C4—H4⋯O1^i^	0.93	2.50	3.421 (5)	171
C11—H11⋯O1^ii^	0.93	2.39	3.296 (5)	165

Symmetry codes: (i) 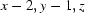; (ii) 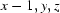.

**Fig. 1.**
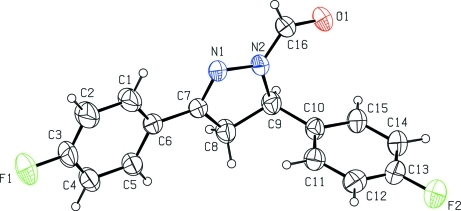
The title molecule with displacement ellipsoids for non-H atoms are drawn at the 30% probability level.

**Fig. 2.**
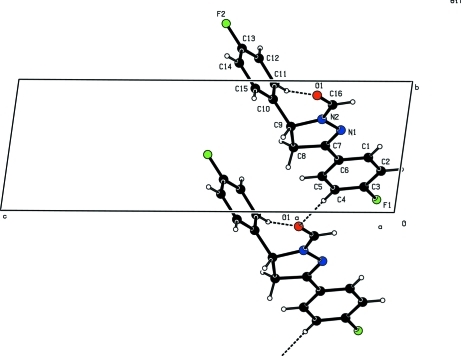
Hydrogen bonding of the title compound viewed along the a axis. Hydrogen bonds are shown as dotted lines (symmetry code: (a) x-2, y-1, z).

## supplementary crystallographic information

### Comment

Pyrazolines have been reported to exhibit a broad spectrum of biological activies such as antitumor, antibacterial, antifungal, antiviral, antiparasitic, anti-tubercular and insecticidal activities (Hes *et al.*, 1978; Manna *et al.*, 2005; Amir *et al.*, 2008). Some of these compounds have also antioxidant, anti-diabetic, anaesthetic and analgesic properties (Sarojini *et al.*, 2010; Regaila *et al.*, 1979). In addition, pyrazolines have played a crucial part in the development of theory in heterocyclic chemistry and also used extensively in organic synthesis (Klimova *et al.*, 1999 and Bhaskarreddy *et al.*, 1997).

The crystal structure of some pyrazoline derivatives *viz*., 3-(4-methylphenyl)-5-[4-(methylthio)phenyl]-4,5-dihydro-1*H*-pyrazole-1-carbaldehyde (Butcher *et al.*, 2007) and 5-(2-hydroxyphenyl)-3-methyl-4,5-dihydro-1*H*-pyrazole-1-carbaldehyde (Cui & Li, 2010) have been reported. In view of the importance of pyrazoline derivatives and in continuation of our work on synthesis of various derivatives of 4,4'-diflouro chalcone (Fun *et al.*, 2010*a*,*b*; Jasinski *et al.*, 2010*a*,*b*; Baktır *et al.*, 2011), the title compound (I) is synthesized and its crystal structure is reported herein.

The molecular structure of the title compound is shown in Fig. 1. The pyrazole ring adopts a slight envelope comformation with the methylene C atom (C8) deviating by 0.114 (3)Å from the mean-plane of the other four atoms (C7/C9/N1/N2 with maximum deviation 0.021 (3)Å for N1). The dihedral angles between the four essentially planar atoms of the pyrazole ring and fluoro-substituted benzene rings are 2.6 (2) and 82.2 (2)°, respectively. The dihedral angle between the two benzene rings is 83.7 (2)°. The crystal packing is stabilized by weak intermolecular C—H···O hydrogen bonds (Fig .2).

### Experimental

A mixture of (2*E*)-1,3-bis(4-fluorophenyl)prop-2-en-1-one (2.44 g, 0.01 mol) and hydrazine hydrate (0.5 ml, 0.01 mol) in 20 ml formic acid was refluxed for 8 h. The reaction mixture was cooled and poured into 50 ml ice-cold water. The precipitate was collected by filtration and purified by recrystallization from ethanol. The single-crystal was grown from DMF by slow evaporation method and yield of the compound was 86%. (m. p.: 408 K).

### Refinement

All H atoms were positioned geometrically [C—H = 0.93 and 0.97 Å] and allowed to ride on their parent C atoms, with *U*_iso_(H) = 1.2*U*_eq_(C). Owing to the large number of weak high-angle reflections, the ratio of observed to unique reflections is low (37%).

### Crystal data

**Table:**

C_16_H_12_F_2_N_2_O	*Z* = 2
*M**_r_* = 286.28	*F*(000) = 296
Triclinic, *P*1	*D*_x_ = 1.418 Mg m^−^^3^
Hall symbol: -P 1	Mo *K*α radiation, λ = 0.71073 Å
*a* = 6.2141 (9) Å	Cell parameters from 1748 reflections
*b* = 6.7802 (8) Å	θ = 2.3–26.3°
*c* = 17.9857 (9) Å	µ = 0.11 mm^−^^1^
α = 96.727 (4)°	*T* = 294 K
β = 90.254 (4)°	Prism, pale yellow
γ = 116.791 (5)°	0.30 × 0.20 × 0.10 mm
*V* = 670.39 (13) Å^3^	

### Data collection

**Table:**

Rigaku R-AXIS RAPID-S diffractometer	2736 independent reflections
Radiation source: Sealed Tube	1011 reflections with *I* > 2σ(*I*)
Graphite Monochromator	*R*_int_ = 0.095
Detector resolution: 10.0000 pixels mm^-1^	θ_max_ = 26.5°, θ_min_ = 3.4°
dtprofit.ref scans	*h* = −7→7
Absorption correction: multi-scan (Blessing, 1995)	*k* = −8→8
*T*_min_ = 0.968, *T*_max_ = 0.989	*l* = −22→22
14070 measured reflections	

### Refinement

**Table:**

Refinement on *F*^2^	Primary atom site location: structure-invariant direct methods
Least-squares matrix: full	Secondary atom site location: difference Fourier map
*R*[*F*^2^ > 2σ(*F*^2^)] = 0.062	Hydrogen site location: inferred from neighbouring sites
*wR*(*F*^2^) = 0.206	H-atom parameters constrained
*S* = 0.94	*w* = 1/[σ^2^(*F*_o_^2^) + (0.0812*P*)^2^] where *P* = (*F*_o_^2^ + 2*F*_c_^2^)/3
2736 reflections	(Δ/σ)_max_ < 0.001
191 parameters	Δρ_max_ = 0.19 e Å^−^^3^
0 restraints	Δρ_min_ = −0.33 e Å^−^^3^

### Special details

**Table:**

Geometry. Bond distances, angles *etc*. have been calculated using the rounded fractional coordinates. All su's are estimated from the variances of the (full) variance-covariance matrix. The cell e.s.d.'s are taken into account in the estimation of distances, angles and torsion angles
Refinement. Refinement on *F*^2^ for ALL reflections except those flagged by the user for potential systematic errors. Weighted *R*-factors *wR* and all goodnesses of fit *S* are based on *F*^2^, conventional *R*-factors *R* are based on *F*, with *F* set to zero for negative *F*^2^. The observed criterion of *F*^2^ > σ(*F*^2^) is used only for calculating -*R*-factor-obs *etc*. and is not relevant to the choice of reflections for refinement. *R*-factors based on *F*^2^ are statistically about twice as large as those based on *F*, and *R*-factors based on ALL data will be even larger.

### Fractional atomic coordinates and isotropic or equivalent isotropic displacement parameters (Å^2^)

**Table:**

	*x*	*y*	*z*	*U*_iso_*/*U*_eq_	
F1	−0.7910 (4)	0.0976 (4)	0.04914 (14)	0.1334 (11)	
F2	0.7113 (5)	1.4342 (4)	0.49400 (14)	0.1284 (11)	
O1	0.9124 (4)	0.8903 (4)	0.23729 (12)	0.0822 (9)	
N1	0.3094 (4)	0.6264 (4)	0.16479 (13)	0.0621 (9)	
N2	0.5069 (4)	0.7082 (4)	0.21640 (12)	0.0621 (9)	
C1	−0.1494 (6)	0.4185 (7)	0.08534 (18)	0.1019 (16)	
C2	−0.3750 (7)	0.3162 (8)	0.0481 (2)	0.120 (2)	
C3	−0.5702 (6)	0.1944 (7)	0.0858 (2)	0.0937 (16)	
C4	−0.5512 (6)	0.1715 (5)	0.1584 (2)	0.0798 (14)	
C5	−0.3241 (6)	0.2737 (5)	0.19570 (18)	0.0718 (12)	
C6	−0.1199 (5)	0.3980 (5)	0.15912 (16)	0.0643 (11)	
C7	0.1190 (5)	0.5070 (5)	0.19872 (15)	0.0583 (11)	
C8	0.1722 (5)	0.4922 (5)	0.27855 (16)	0.0728 (12)	
C9	0.4427 (5)	0.6519 (5)	0.29283 (15)	0.0650 (11)	
C10	0.5095 (5)	0.8590 (5)	0.34783 (15)	0.0637 (11)	
C11	0.3760 (6)	0.9769 (6)	0.34948 (17)	0.0713 (11)	
C12	0.4417 (7)	1.1706 (6)	0.39825 (19)	0.0820 (16)	
C13	0.6424 (7)	1.2450 (6)	0.4449 (2)	0.0873 (16)	
C14	0.7786 (7)	1.1343 (6)	0.44657 (19)	0.0870 (14)	
C15	0.7124 (6)	0.9408 (6)	0.39679 (17)	0.0774 (14)	
C16	0.7313 (6)	0.8185 (5)	0.19475 (18)	0.0696 (12)	
H1	−0.01520	0.50270	0.06000	0.1230*	
H2	−0.39330	0.33040	−0.00210	0.1440*	
H4	−0.68760	0.08870	0.18310	0.0960*	
H5	−0.30840	0.25880	0.24590	0.0860*	
H8A	0.13880	0.34140	0.28510	0.0870*	
H8B	0.07740	0.53940	0.31210	0.0870*	
H9	0.52740	0.57050	0.30870	0.0780*	
H11	0.23920	0.92430	0.31700	0.0850*	
H12	0.35050	1.24820	0.39910	0.0990*	
H14	0.91270	1.18720	0.48020	0.1040*	
H15	0.80540	0.86500	0.39630	0.0930*	
H16	0.75090	0.84190	0.14480	0.0830*	

### Atomic displacement parameters (Å^2^)

**Table:**

	*U*^11^	*U*^22^	*U*^33^	*U*^12^	*U*^13^	*U*^23^
F1	0.0603 (14)	0.164 (2)	0.142 (2)	0.0304 (15)	−0.0376 (13)	−0.0172 (17)
F2	0.1089 (18)	0.1004 (17)	0.133 (2)	0.0232 (15)	0.0018 (15)	−0.0407 (15)
O1	0.0474 (13)	0.0936 (18)	0.0902 (17)	0.0222 (13)	−0.0112 (12)	−0.0025 (13)
N1	0.0507 (15)	0.0728 (18)	0.0553 (14)	0.0242 (14)	−0.0057 (12)	−0.0030 (12)
N2	0.0451 (15)	0.0734 (18)	0.0576 (14)	0.0203 (14)	−0.0060 (11)	−0.0010 (12)
C1	0.059 (2)	0.159 (4)	0.061 (2)	0.027 (2)	−0.0034 (17)	0.011 (2)
C2	0.070 (3)	0.187 (5)	0.074 (2)	0.037 (3)	−0.015 (2)	0.001 (3)
C3	0.052 (2)	0.105 (3)	0.107 (3)	0.028 (2)	−0.022 (2)	−0.016 (2)
C4	0.051 (2)	0.072 (2)	0.104 (3)	0.0191 (18)	−0.0005 (18)	0.004 (2)
C5	0.059 (2)	0.069 (2)	0.080 (2)	0.0242 (18)	−0.0004 (17)	0.0038 (17)
C6	0.053 (2)	0.075 (2)	0.0613 (18)	0.0286 (18)	−0.0030 (14)	−0.0025 (15)
C7	0.0502 (19)	0.066 (2)	0.0554 (17)	0.0254 (16)	0.0004 (14)	0.0000 (14)
C8	0.063 (2)	0.074 (2)	0.068 (2)	0.0199 (18)	−0.0071 (15)	0.0074 (16)
C9	0.061 (2)	0.070 (2)	0.0583 (17)	0.0253 (17)	−0.0090 (14)	0.0073 (15)
C10	0.0568 (19)	0.072 (2)	0.0541 (17)	0.0232 (17)	−0.0080 (14)	0.0042 (15)
C11	0.065 (2)	0.084 (2)	0.0618 (18)	0.032 (2)	−0.0040 (15)	0.0066 (17)
C12	0.085 (3)	0.086 (3)	0.075 (2)	0.040 (2)	0.0065 (19)	0.0061 (19)
C13	0.082 (3)	0.077 (3)	0.080 (2)	0.022 (2)	0.002 (2)	−0.0147 (19)
C14	0.069 (2)	0.095 (3)	0.078 (2)	0.026 (2)	−0.0154 (18)	−0.011 (2)
C15	0.064 (2)	0.091 (3)	0.066 (2)	0.029 (2)	−0.0137 (16)	−0.0044 (18)
C16	0.050 (2)	0.081 (2)	0.072 (2)	0.0268 (18)	−0.0002 (16)	0.0009 (17)

### Geometric parameters (Å, °)

**Table:**

F1—C3	1.352 (5)	C10—C15	1.385 (5)
F2—C13	1.358 (4)	C11—C12	1.380 (5)
O1—C16	1.225 (4)	C12—C13	1.356 (6)
N1—N2	1.390 (4)	C13—C14	1.363 (6)
N1—C7	1.299 (4)	C14—C15	1.388 (5)
N2—C9	1.478 (4)	C1—H1	0.9300
N2—C16	1.337 (5)	C2—H2	0.9300
C1—C2	1.379 (6)	C4—H4	0.9300
C1—C6	1.370 (4)	C5—H5	0.9300
C2—C3	1.360 (6)	C8—H8A	0.9700
C3—C4	1.344 (5)	C8—H8B	0.9700
C4—C5	1.387 (5)	C9—H9	0.9800
C5—C6	1.388 (5)	C11—H11	0.9300
C6—C7	1.461 (5)	C12—H12	0.9300
C7—C8	1.497 (4)	C14—H14	0.9300
C8—C9	1.534 (5)	C15—H15	0.9300
C9—C10	1.506 (4)	C16—H16	0.9300
C10—C11	1.386 (5)		
			
N2—N1—C7	107.5 (2)	C13—C14—C15	118.4 (4)
N1—N2—C9	113.9 (2)	C10—C15—C14	120.9 (4)
N1—N2—C16	120.5 (2)	O1—C16—N2	123.4 (3)
C9—N2—C16	125.6 (3)	C2—C1—H1	119.00
C2—C1—C6	121.0 (4)	C6—C1—H1	119.00
C1—C2—C3	119.0 (3)	C1—C2—H2	120.00
F1—C3—C2	118.6 (3)	C3—C2—H2	121.00
F1—C3—C4	119.2 (4)	C3—C4—H4	121.00
C2—C3—C4	122.2 (4)	C5—C4—H4	121.00
C3—C4—C5	118.7 (3)	C4—C5—H5	120.00
C4—C5—C6	120.9 (3)	C6—C5—H5	120.00
C1—C6—C5	118.1 (3)	C7—C8—H8A	111.00
C1—C6—C7	121.1 (3)	C7—C8—H8B	111.00
C5—C6—C7	120.8 (3)	C9—C8—H8A	111.00
N1—C7—C6	120.7 (3)	C9—C8—H8B	111.00
N1—C7—C8	113.8 (3)	H8A—C8—H8B	109.00
C6—C7—C8	125.5 (3)	N2—C9—H9	109.00
C7—C8—C9	103.6 (2)	C8—C9—H9	109.00
N2—C9—C8	100.7 (2)	C10—C9—H9	109.00
N2—C9—C10	111.1 (2)	C10—C11—H11	119.00
C8—C9—C10	116.5 (3)	C12—C11—H11	119.00
C9—C10—C11	121.5 (3)	C11—C12—H12	121.00
C9—C10—C15	120.4 (3)	C13—C12—H12	121.00
C11—C10—C15	118.1 (3)	C13—C14—H14	121.00
C10—C11—C12	121.4 (4)	C15—C14—H14	121.00
C11—C12—C13	118.4 (4)	C10—C15—H15	120.00
F2—C13—C12	119.9 (4)	C14—C15—H15	120.00
F2—C13—C14	117.3 (4)	O1—C16—H16	118.00
C12—C13—C14	122.8 (4)	N2—C16—H16	118.00
			
C7—N1—N2—C9	−4.2 (3)	C5—C6—C7—N1	−178.8 (3)
C7—N1—N2—C16	173.1 (3)	C5—C6—C7—C8	2.3 (5)
N2—N1—C7—C8	−1.3 (4)	C1—C6—C7—N1	0.1 (5)
N2—N1—C7—C6	179.7 (3)	C6—C7—C8—C9	−175.2 (3)
C16—N2—C9—C10	66.3 (4)	N1—C7—C8—C9	5.9 (4)
C16—N2—C9—C8	−169.7 (3)	C7—C8—C9—N2	−7.3 (3)
C9—N2—C16—O1	−1.5 (5)	C7—C8—C9—C10	112.8 (3)
N1—N2—C9—C8	7.4 (3)	C8—C9—C10—C11	−40.0 (4)
N1—N2—C9—C10	−116.5 (3)	C8—C9—C10—C15	142.1 (3)
N1—N2—C16—O1	−178.4 (3)	N2—C9—C10—C15	−103.5 (3)
C2—C1—C6—C7	−179.8 (4)	N2—C9—C10—C11	74.4 (4)
C6—C1—C2—C3	0.3 (7)	C9—C10—C15—C14	178.6 (3)
C2—C1—C6—C5	−0.9 (6)	C9—C10—C11—C12	−177.9 (3)
C1—C2—C3—C4	0.5 (7)	C15—C10—C11—C12	0.0 (5)
C1—C2—C3—F1	179.0 (4)	C11—C10—C15—C14	0.6 (5)
F1—C3—C4—C5	−179.3 (3)	C10—C11—C12—C13	0.3 (5)
C2—C3—C4—C5	−0.8 (6)	C11—C12—C13—C14	−1.3 (6)
C3—C4—C5—C6	0.2 (5)	C11—C12—C13—F2	−179.5 (3)
C4—C5—C6—C1	0.6 (5)	F2—C13—C14—C15	−179.8 (3)
C4—C5—C6—C7	179.5 (3)	C12—C13—C14—C15	1.9 (6)
C1—C6—C7—C8	−178.8 (3)	C13—C14—C15—C10	−1.6 (5)

### Hydrogen-bond geometry (Å, °)

**Table:**

*D*—H···*A*	*D*—H	H···*A*	*D*···*A*	*D*—H···*A*
C4—H4···O1^i^	0.93	2.50	3.421 (5)	171
C11—H11···O1^ii^	0.93	2.39	3.296 (5)	165

Symmetry codes: (i) *x*−2, *y*−1, *z*; (ii) *x*−1, *y*, *z*.
